# Mitochondrial DNA depletion induces innate immune dysfunction rescued by IFN-γ

**DOI:** 10.1016/j.jaci.2017.04.048

**Published:** 2017-11

**Authors:** John D. Widdrington, Aurora Gomez-Duran, Jannetta S. Steyn, Angela Pyle, Marie-Helene Ruchaud-Sparagano, Jonathan Scott, Simon V. Baudouin, Anthony J. Rostron, John Simpson, Patrick F. Chinnery

**Affiliations:** a Institute of Cellular Medicine, Newcastle University, Newcastle upon Tyne, United Kingdom; b Institute of Genetic Medicine, Newcastle University, Newcastle upon Tyne, United Kingdom; c Department of Anaesthesia, Royal Victoria Infirmary, Newcastle upon Tyne, United Kingdom; d Medical Research Council Mitochondrial Biology Unit, Cambridge Biomedical Campus, Cambridge, United Kingdom; e Department of Clinical Neurosciences, University of Cambridge, Cambridge, United Kingdom; f Bioinformatics Support Unit, Faculty of Medical Sciences, Newcastle University, Newcastle upon Tyne, United Kingdom

To the Editor:

Sepsis is a clinical syndrome with increasing incidence and mortality in which a systemic inflammatory response is triggered by infection. The clinical outcome of sepsis is primarily determined by the host response; in particular, monocyte deactivation plays a key role in sepsis-induced immune suppression and contributes to mortality.^1^, ^2^ While the underlying mechanisms of monocyte deactivation are not understood, there is increasing evidence that mitochondrial dysfunction contributes to the pathogenesis of sepsis. Monocytes from sepsis patients have impaired mitochondrial respiration and depletion of mitochondrial DNA (mtDNA). These findings correlate with the severity of the illness,^3^, ^4^, ^5^, ^6^ but it is unclear whether the mitochondrial defects lead to the immune deactivation of blood monocytes or occur simply as a consequence of sepsis. To address this issue, we studied the effects of reducing mtDNA levels on immune function in THP-1 cells, a human monocyte cell line.

Treatment of THP-1 cells with 50 ng/mL ethidium bromide for 8 weeks generated ρ0 cells lacking mtDNA (Fig 1, *A*) without adverse effects on cell viability (see Fig E1, *A* and the Methods in this article's Online Repository at www.jacionline.org). This completely suppressed mtDNA-encoded MT-CO1 protein levels and cytochrome c oxidase activity, without affecting nuclear-encoded SDHA expression or mitochondrial mass, measured by citrate synthase activity (Fig E1, *B-D*). The ρ0 cells had a blunted TNF-α response to treatment with 100 ng/mL LPS for 4 hours (Fig 1, *B*), consistent with monocyte deactivation. Repeating the experiments with short-interfering RNA ([siRNA], 30 nmol/L for 8 days) silencing the expression of mitochondrial transcription factor A (TFAM), a major component of the mitochondrial nucleoid that regulates mtDNA replication and gene expression (Fig 1, *C*), also suppressed mtDNA levels (Fig 1, *D*), reduced mitochondrial-encoded proteins and oxygen consumption (see Fig E2, *C* and *D* in this article's Online Repository at www.jacionline.org), and impaired the TNF-α response to LPS (Fig 1, *E*). While the *TFAM* siRNA-transfected THP-1 cells also had a reduced ability to phagocytose the gram-negative bacterium *Escherichia coli* (Fig E2, *E*), there was not a global downregulation of immunity as LPS-induced IL-8 production was unaltered (Fig 1, *E*). The effects of mtDNA depletion were partially reversed after removal of the siRNA (Fig 1, *F-H* and Fig E2, *F-H*).

**Fig 1:**
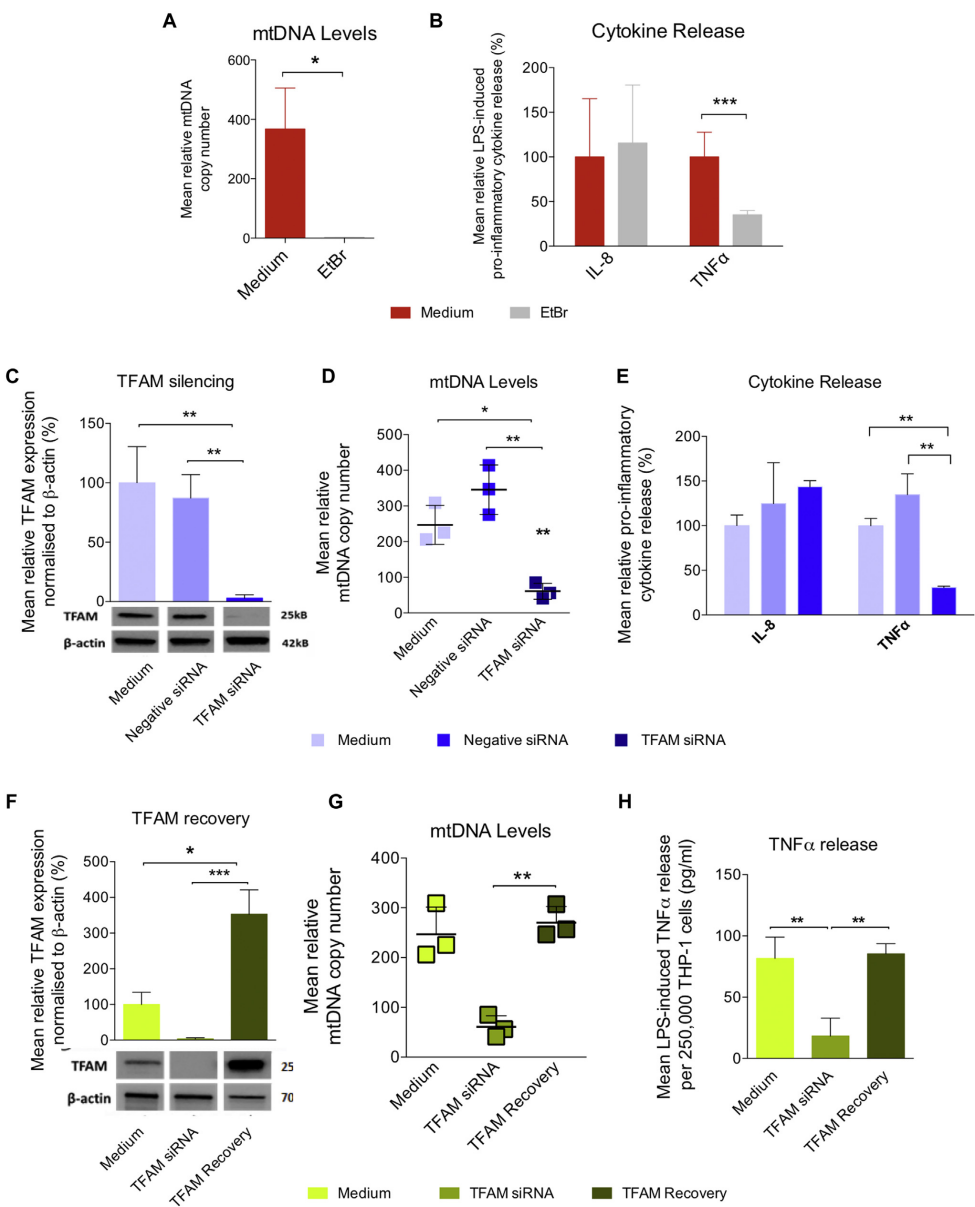
MtDNA depletion and reversible impaired immune functions in THP-1 cells. **A** and **B,** Treatment with 50 ng/mL ethidium bromide (EtBr) for 8 weeks. **A,** MtDNA levels. **B,** LPS-induced TNF-α and IL-8 release. **C-E,** Transfection with 30 nmol/L negative or *TFAM* siRNA for 8 days. **C,** TFAM protein relative to β-actin. **D,** MtDNA levels. **E,** LPS-induced TNF-α and IL-8 release. **F-H,** TFAM recovery 8 days after removal of *TFAM* siRNA. **F,** TFAM protein relative β-actin. **G,** MtDNA levels. **H,** LPS-induced TNF-α and IL-8 release. All experiments are presented as means ± SD of 3 to 4 independent biological replicates. *P < .05, **P < .01, and ***P < .001.

To determine the mechanism linking mtDNA depletion with impaired immune function, we performed whole transcriptome RNA-Seq before and after *TFAM* siRNA transfection (Fig 2, *A* and Fig E3 in the Online Repository at www.jacionline.org). There were 1389 differential expressed genes in *TFAM* siRNA-transfected THP-1 cells compared with control cells (Fig 2, *A* and *B*, see Data File E1 in this article's Online Repository at www.jacionline.org). Ingenuity Pathway Analysis (IPA) of the gene ontology showed suppression of key innate immune signaling pathways, including interferon and TREM1 signaling (Fig 2, *C*, and see Fig E3 and Table E1 in this article's Online Repository at www.jacionline.org). Following 4 hours' treatment with 100 ng/mL LPS, we observed a consistent upregulation of inflammatory genes (log fold-change [LogFC] > 1.5) (Fig E3, *D*). Gene ontology analysis showed that mtDNA depletion was associated with a significant downregulation of multiple signaling pathways involved in pathogen recognition following exposure to LPS (Fig E3, *E-H*, Table E2). Thus, the mtDNA depletion induced by *TFAM* siRNA blunts the immune response of THP-1 cells to LPS through known innate immune pathways. These findings were validated in independent experiments using quantitative RT-PCR; mtDNA depletion blunted the LPS-induced upregulation for key genes encoding cell surface receptors (*TLR4, TREM1*), proinflammatory cytokines (*IL1B, TNF*), interferon signaling molecules (*IFIT1, IFITM1*), and inflammatory mediators (*MYD88, STAT1*) (Fig 2, *D-G*). TLR-4 expression, measured by flow cytometry, was significantly decreased following mtDNA depletion (Fig 2, *H*), providing a potential explanation for the blunted immune response in THP-1 cells lacking mtDNA.

**Fig 2:**
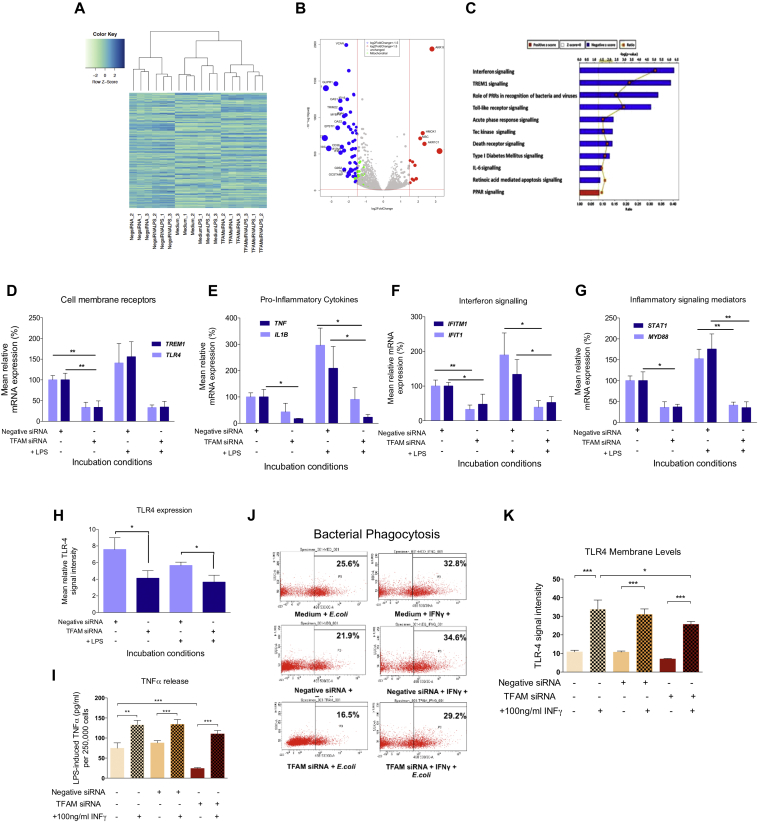
Transcriptomic response and immune dysfunction in *TFAM* siRNA-induced mtDNA-depleted THP-1 cells is rescued by IFN-γ treatment. **A,** Hierarchical clustering for the 3000 most expressed genes in all the samples used for RNA-Seq. **B,** Volcano plot of differentially expressed genes between control and siRNA cells (dot size proportional to LogFC >1.5 red [upregulated], blue [downregulated], green = the mitochondrial genes). **C,** Altered canonical signaling pathways with siRNA. **D-G,** Quantitative PCR validation of key genes. **H,** Cell surface expression of TLR-4. **I,** LPS-induced TNF-α release after treatment with 100 ng/mL IFN-γ for the final 24 hours of the transfection period. **J,** Phagocytosis of *E coli*. **K,** Phagocytosis of *E coli*. Data are presented as means ± SD of 3 independent biological replicates; *P < .05, **P < .01, and ***P < .001. *iNOS*, Inducible nitric oxide synthase; *IRF*, interferon regulatory factor; *PI3K*, phoshoinositide-3-kinase; *RIG1*, retinoic acid-inducible gene-1; *TSP-1*, thrombospondin-1.

IFN-γ has been shown to reverse immune deactivation in septic monocytes^7^ and is a monocyte activator that stimulates TLR-4 expression through the interferon signaling pathways that are downregulated with mtDNA depletion. Treating mtDNA-depleted THP-1 cells with 100 ng/mL recombinant human IFN-γ in the final 24 hours of the 8-day siRNA transfection period had no adverse effects on THP-1 cell viability (see Fig E4, *A* in the Online Repository at www.jacionline.org), but increased both LPS-induced TNF-α release (Fig 2, *I*) and the capacity to phagocytose *E coli* (Fig 2, *J* and Fig E4, *B*). IFN-γ treatment increased cell surface expression of TLR-4 in all experimental conditions (Fig 2, *K* and Fig E4, *C*).

Using 2 independent methods to induce mtDNA depletion, we show that mtDNA depletion can reversibly impair innate immune responses in THP-1 cells. In particular, we identify a significant inhibition of TNF-α production in response to LPS, thus reproducing the key phenotypic marker of immune deactivation in monocytes from patients with sepsis. The mtDNA depletion also inhibits interferon and pattern-recognition receptor-mediated signaling and decreased cell surface expression of TLR-4, changes that would fundamentally impair the responses of THP-1 cells to LPS and gram-negative bacteria.

How can we explain the transcriptional changes we observed following mtDNA depletion? Mitochondrial abundance and mtDNA levels are tightly regulated in response to cellular energetic demands, and mtDNA depletion leads to a bioenergetic defect of OXPHOS and a reduction in ATP production. This could have several consequences. First, in cell lines from patients with rare inherited mtDNA mutations, the biochemical defect activates a retrograde signaling response from the mitochondria to the nucleus that alters the transcription of several genes known to be involved in immune activation. Linked to this there may be compensatory mitochondrial biogenesis, including the activation of peroxisome proliferator activated receptor (PPAR) signaling, similar to our observation in mtDNA-depleted THP-1 cells (Fig 2, *C* and Fig E3). Increased PPAR signaling has been associated with a shift to an anti-inflammatory phenotype in animal models of sepsis.^8^ Finally, the shift from oxidative to glycolytic metabolism in mtDNA-depleted THP-1 cells could produce changes in gene expression and immune phenotype. However, in macrophages, a shift to glycolytic metabolism has been associated with the adoption of a proinflammatory phenotype, with anti-inflammatory macrophages rather having enhanced OXPHOS activity.^8^

During severe sepsis, intense on-going mtDNA damage and mitochondrial dysfunction could overwhelm the capacity for mitochondrial biogenesis, leading to a gradual decline in mtDNA levels over time. Our data suggest that this may contribute to monocyte immune deactivation, which is associated with adverse clinical outcomes and could be reversed by IFN-γ. Our observations were made on a transformed human monocyte line and focused on TLR-4 specific mechanisms. If confirmed in human monocytes this would provide new opportunities to treat sepsis.

## Figures and Tables

**Fig E1:**
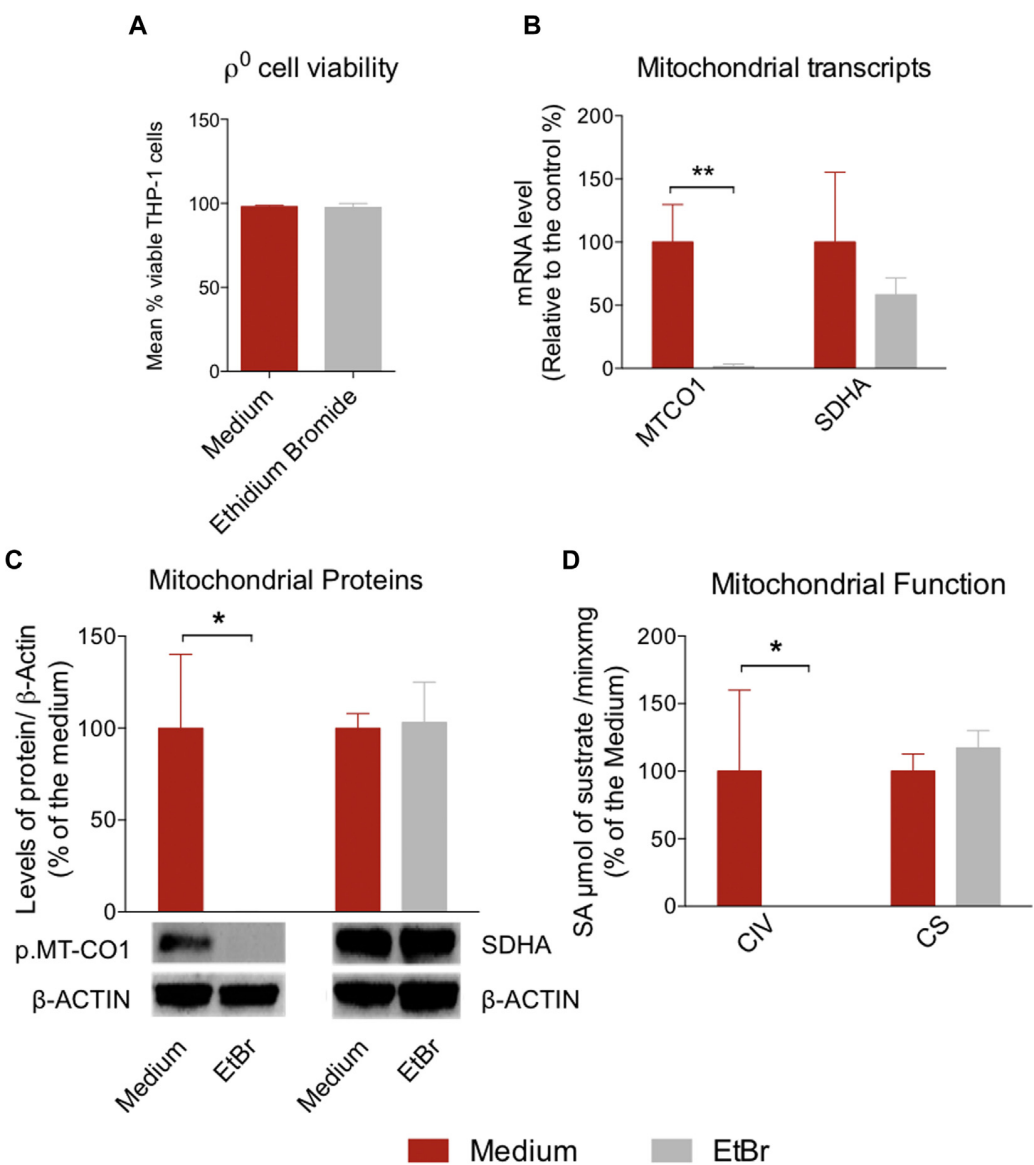
Effects of mtDNA depletion on THP-1 cells. THP-1 cells were treated with 50 ng/mL EtBr for 8 weeks. **A,** Cell viability. **B,** Mitochondrial transcripts. **C,** Mitochondrial proteins relative to β-actin. **D,** Spectrophotometric measurements of isolated respiratory enzyme activity in cellular homogenates for complex IV (CIV) and citrate synthase (CS) activity. All experiments had 3 independent biological replicates and are presented as means ± SD. *P < .05 and **P < .01.

**Fig E2:**
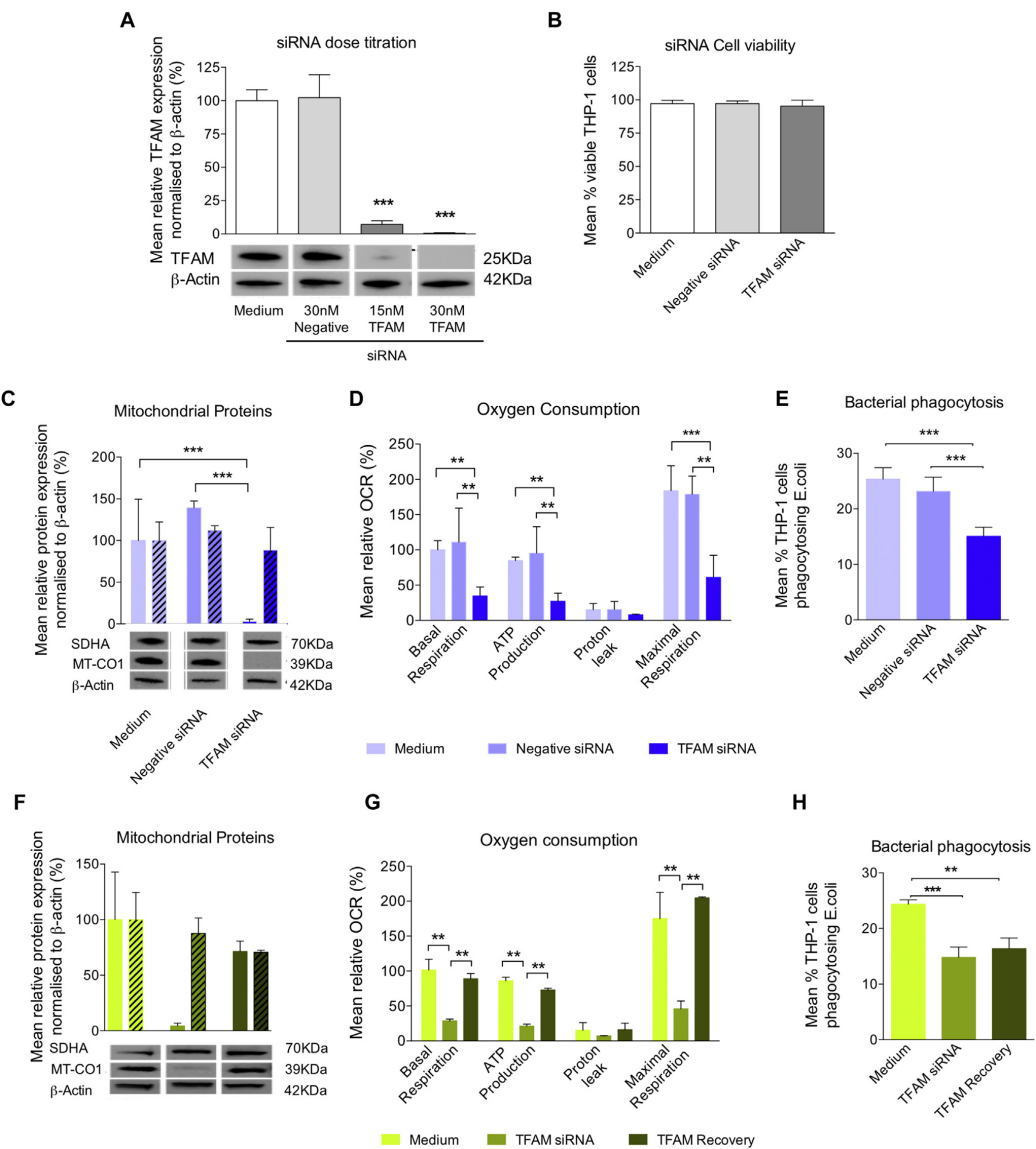
MtDNA depletion and impaired immune functions and subsequent recovery in THP-1 cells following transfection with *TFAM* siRNA. **A-G,** Transfection with *TFAM* siRNA. **A,** TFAM proteins levels relative to β-actin during titration of *TFAM* siRNA, showing optimal knockdown of TFAM protein after transfection of THP-1 cells with 30 nmol/L siRNA for 8 days. **B,** Cell viability. **C,** Cell proliferation. **D,** The levels of the MT-CO1 and SDHA proteins relative to β-actin. **D,** OCR for different aspects of mitochondrial respiration and respiratory profile. **E,** Phagocytosis of *E coli*. Recovery 8 days after removal of *TFAM* siRNA. **F,** Levels of the MT-CO1 and SDHA proteins relative to β-actin. **G,** Oxygen consumption for different aspects of mitochondrial respiration. **H**, Bacterial phagocytosis. All experiments were carried out on 3 to 4 independent biological replicates and are presented as means ± SD. **P < .01 and ***P < .001.

**Fig E3:**
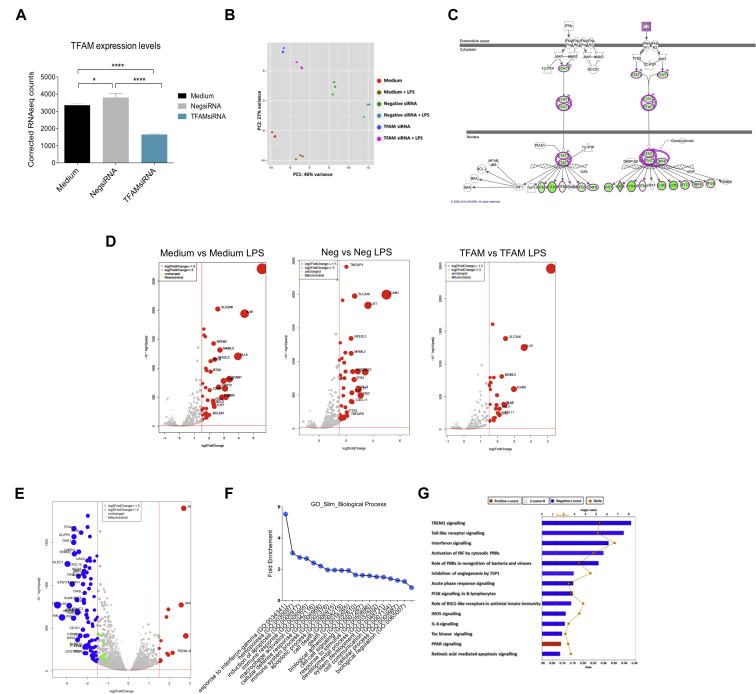
Canonical signaling pathways in mtDNA-depleted THP-1 cells with and without LPS treatment. THP-1 cells were incubated in growth medium or transfected with 30 nmol/L of negative or *TFAM* siRNA for 8 days. After a final incubation with 100 ng/mL LPS or medium for 4 hours, gene expression was assessed by RNA-Seq. **A,** Levels of transcripts for *TFAM*. **B,** Principal component analysis plot. **C,** The effect of transfection with *TFAM* siRNA on the interferon signaling pathway extracted from IPA (downregulated genes are highlighted in green and upregulated genes are highlighted in red). **D,** Volcano plot showing differentially expressed genes in each experimental condition following treatment with LPS. **E,** Volcano plot comparing the LPS response of *TFAM* siRNA-transfected cells to negative control siRNA transfected cells. (In the volcano plots, differentially expressed genes are highlighted in gray, genes with LogFC >1.5 are represented in red [upregulated] or blue [downregulated], while mitochondrial genes are highlighted in green. The size of the dots is proportional to the LogFC.) **F,** Gene Slim Ontology analysis carried out by Gene Ontology Consortium Fold Change enrichment for pathways with *P* < .05. **G,** IPA analysis of the canonical signaling pathways significantly affected by the differential expression of genes after treatment with LPS in *TFAM* siRNA-transfected THP-1 cells compared with negative siRNA-transfected cells. The data were filtered for Benjamini-Hochberg multiple testing correction *P*-value <.05 and *z*-score >±2. *P < .05 and ****P < .0001.

**Fig E4:**
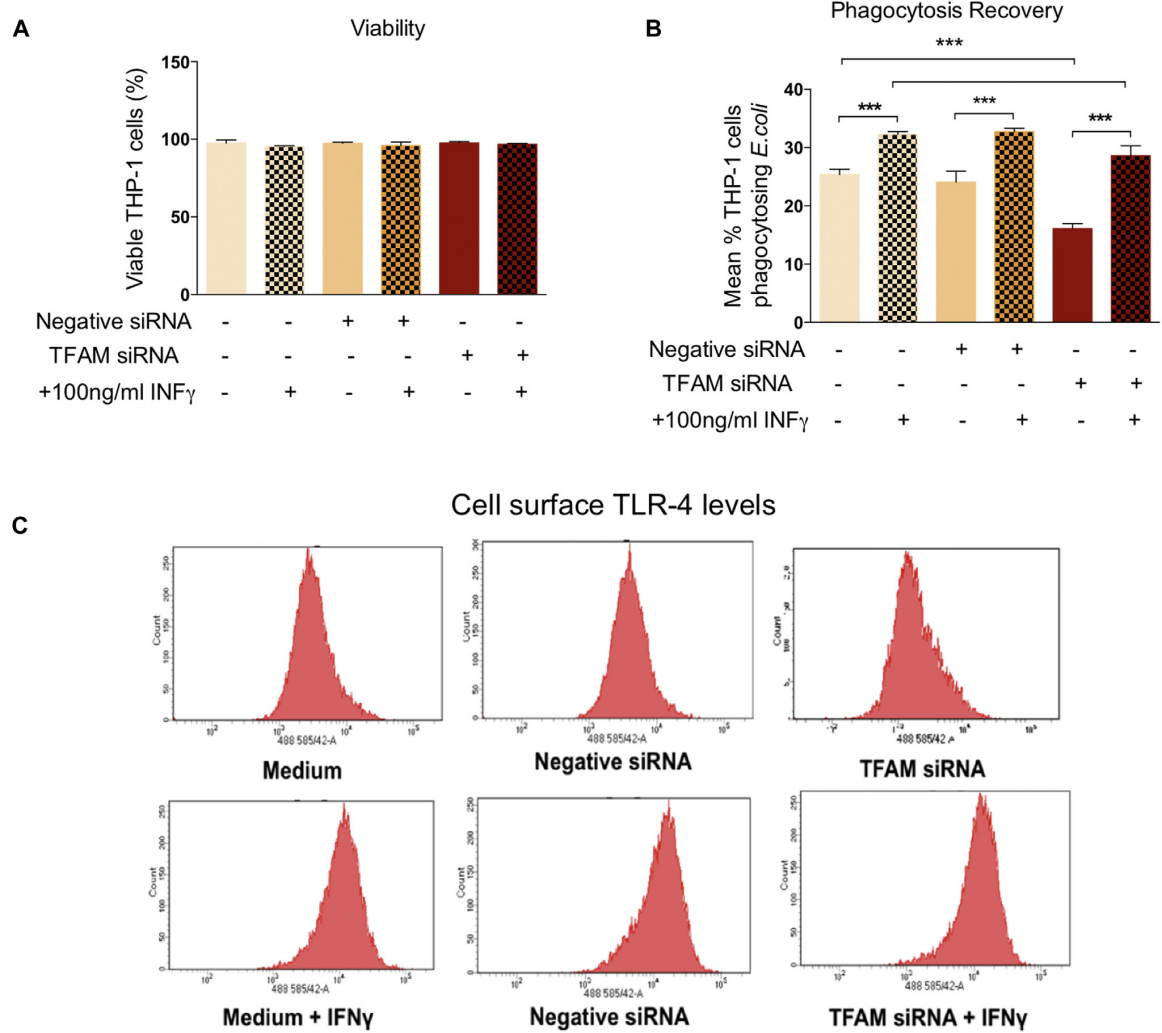
Treatment with IFN-γ increases TLR-4 expression and restores immune functions in THP-1 cells with mtDNA depletion following transfection with *TFAM* siRNA. THP-1 cells were treated with 100 ng/mL recombinant human IFN-γ or medium for the final 24 hours of an 8-day transfection with negative control or *TFAM* siRNA, or incubation with growth medium. **A,** Cell viability. **B,** Phagocytosis of *E coli*. **C,** Cell surface expression of TLR-4. All experiments were carried out on 3 independent biological replicates and are presented as means ± SD. ***P < .001.

**Table E1:** Details of the 5 signaling pathways most significantly affected by transfection with *TFAM* siRNA

Function	*z*-score	*P*-value	Genes with altered expression		
			Proportion of genes in pathway	Upregulated	Downregulated
Interferon signaling					
Cellular immune response Cytokine signaling	−3.16	4.9 × 10^−7^	11/34	—	*IFI35, IFNB1, IFIT1, IFITM1, IFITM2, IFITM3, IFIT3, IRF9, OAS1, STAT2, STAT1*
TREM1 signaling					
Cellular immune response Cytokine response	−3.50	7.9 × 10^−7^	16/75	*MPO*	*CCL3, CD83, CIITA, IL1B, ITGAX, MYD88, NLRC4, NLRP12, TLR1, TLR3, TLR6, TLR7, TNF, TREM1, TYROBP*
Role of pattern recognition receptors in recognition of bacteria and viruses					
Cellular immune response Pathogen-influenced signaling	−3.74	5.5 × 10^−6^	20/127	*IL12A*	*C3AR1, C5AR1, DDX58, EIF2AK2, IFIH1, IFNB1, IL1B, IRF7, MYD88, NLRC4, OAS1, OAS2, OAS3, PTX3, TLR1, TLR3, TLR6, TLR7, TNF*
Toll-like receptor signaling					
Apoptosis Cellular immune response Humoral immune response Pathogen-influenced signaling	−2.33	1.7 × 10^−5^	14/74	*IL12A, PPARA*	*EIF2AK2, FOS, IL1B, IL1RN, MYD88, NFKBIA, TLR1, TLR3, TLR6, TLR7, TNF, TNFAIP3*
Acute phase response					
Cytokine signaling	−2.67	5.2 × 10^−3^	17/169	*FTL, HMOX1, ORM1, ORM2, SOCS2*	*A2M, AGT, CEBPB, FOS, IKBKE, IL1B, IL1RN, MYD88, NFKBIA, SERPINE1, SOCS3, TNF*

**Table E2:** Details of the 5 pathways most significantly affected by the altered transcriptomic response to LPS following transfection with *TFAM* siRNA

Function	*z*-score	*P*-value	Genes with altered expression		
			Proportion	Upregulated	Downregulated
TREM1 signaling					
Cellular immune response Cytokine response	−4.15	6.3 × 10^−9^	21/75	*MPO*	*CCL2, CCL3, CD40, CD83, CIITA, ICAM1, IL1B, ITGAX, MYD88, NLRC4, NLRP12, NOD2, TLR1, TLR3, TLR4, TLR6, TLR7, TNF, TREM1, TYROBP*
Toll-like receptor signaling					
Apoptosis Cellular immune response Humoral immune response Pathogen-influenced signaling	−2.13	2.8 × 10^−8^	20/74	*ELK1, IL12A, MAP3K14, PPARA, TRAF4*	*EIF2AK2, FOS, IL1B, IL1RN, IRAK2, JUN, MYD88, NFKBIA, TLR1, TLR3, TLR4, TLR6, TLR7, TNF, TNFAIP3*
Interferon signaling					
Cellular immune response Cytokine signaling	−3.32	7.2 × 10^−7^	12/34	—	*IFI35, IFIT1, IFIT3, IFITM1, IFITM3, IFNB1, IRF9, MX1, OAS1, STAT2, STAT1, TAP1*
Activation of interferon regulatory factors by cytosolic pattern recognition receptors					
Cellular immune response	−2.00	2.1 × 10^−6^	12/34	—	*ADAR, CD40, DDX58, DHX58, IFIH1, IFIT2, IFNB1, IKBKE, IRF7, IRF9, ISG15, JUN, NFKBIA, STAT1, STAT2, TNF*
Role of pattern recognition receptors in recognition of bacteria and viruses					
Cellular immune response Pathogen-influenced signaling	−3.74	5.5 × 10^−6^	20/127	*IL12A*	*C3, C3AR1, C5AR1, DDX58, EIF2AK2, IFIH1, IFNB1, IL1B, IRF7, MYD88, NLRC4, NOD2, OAS1-3, PTX3, TLR1, TLR3, TLR4, TLR6, TLR7, TNF*

## Methods

### Cell culture

THP-1 cells (TIB-202; ATCC, Manassas, Va) were maintained at a concentration of <1 × 10^6^ cells/mL in RPMI 1640 medium supplemented with 10% FCS (subsequently termed growth medium) and contamination with mycoplasma was periodically excluded. Cell viability was determined by exclusion of 0.4% trypan blue (Sigma-Aldrich, St Louis, Mo), propidium iodide or 7-aminoactinomycin-D. All reagents were obtained from Thermo Fisher Scientific unless otherwise stated.

### Generation of ρ0 THP-1 cells

THP-1 cells were incubated in RPMI 1640 medium supplemented with 50 ng/mL ethidium bromide, 110 μg/mL sodium pyruvate, 50 μg/mL uridine, 2 mmol/L L-glutamine, and 10% FCS (all final concentrations) for 8 weeks.

### RNA interference

THP-1 cells were transfected with Silencer Select *TFAM* siRNA (s14001, sense—GAAGAGAUAAGCAGAUUUAtt, antisense—UAAAUCUGCUUAUCUCUUCtt) or Silencer Select Negative Control siRNA number 1 (Thermo Fisher Scientific, Waltham, Mass) using the Lipofectamine RNAiMAX transfection reagent (Invitrogen, Thermo Fisher Scientific) and following the manufacturers' protocols. The transfection was repeated every 48 hours for 8 days. The effect of IFN-γ was determined by treating THP-1 cells with 100 ng/mL recombinant human IFN-γ (R&D Systems, Minneapolis, Minn) for the final 24 hours of the 8 day siRNA transfection.

### Detection of cytokines

We seeded 2.5 × 10^5^ THP-1 cells in 500 μL growth medium per well onto a 24-well plate (Grenier Bio-One, Monroe, NC) and incubated for 4 hours at 37°C ± 100 ng/mL LPS from *E coli* O26:B6 (Sigma-Aldrich). Subsequently, the release of TNF-α and IL-8 in supernatant samples was measured by ELISA using Novex Human Antibody Pair kits (Invitrogen) and following the manufacturer's protocol.

### Phagocytosis of *E coli*

Serum-opsonized fluorescein-labelled *E coli* K-12 strain were incubated with THP-1 cells at a multiplicity of infection of 10:1 for 1 hour at 37°C. After washing and quenching extracellular fluorescence through the addition of 0.1% trypan blue, the proportion of cells internalizing bacteria was then measured using the FACSCanto II flow cytometer (BD Biosciences, San Jose, Calif).

### MtDNA copy number

DNA was extracted from cell pellets using the DNeasy blood and tissue kit (Qiagen, Hilden, Germany). The relative mtDNA copy number was determined by comparing the level of the mtDNA-encoded *MT-ND1* gene (primers: F—ACGCCATAAAACTCTTCACCAAAG, R—GGGTTCATAGTAGAAGAGCGATGG) to that of the nuclear reference gene *B2M* (primers: F—CACTGAAAAAGATGAGTATGCC, R—AACATTCCCTGACAATCCC) by quantitative RT-PCR using the SYBR Green technique and the MyiQ PCR machine (both BioRad Laboratories, Hercules, Calif).^E1^

### Immunoblotting

THP-1 cells were lysed using a lysis buffer containing 1% Triton X and 1 mmol/L of the protease inhibitor phenylmethanesulfonyl fluoride (both Sigma-Aldrich) and the protein concentration in the lysates determined by Bradford assay. Equal amounts of protein were separated on the basis of size by SDS-PAGE, transferred onto polyvinylidene fluoride membranes and blotted with different antibodies. Signal intensity was assessed after addition of an enhanced chemiluminescent substrate using the MultiSpectral Imaging System (UVP, Upland, Calif). In addition to the antimouse Ig-HRP (0260) secondary antibody from Dako (Agilent, Santa Clara, Calif), the following mouse antihuman antibodies were used: β-actin (ab8226), MTCO1 (ab14705), and SDHA (ab14715) from Abcam (Cambridge, UK) and TFAM (NBP1-71648) from Novus Biological (Littleton, Colo).

### Oxygen consumption

Oxygen consumption for different aspects of mitochondrial respiration was measured using the Mito Stress kit and the Seahorse XF96^e^ Extracellular Flux analyzer (both Seahorse Biosciences, Chicopee, Mass) as previously described.^E2^ In each well 0.8 × 10^5^ THP-1 cells were seeded in 175 μL of an assay medium, consisting of modified Eagle medium supplemented with 11.1 mmol/L D-glucose and 2 mmol/L L-glutamine and adjusted to pH 7.0. Oxygen consumption rate (OCR) was measured at baseline and following the sequential addition of 1 μmol/L oligomycin (a complex V inhibitor), 0.5 μmol/L then 1 μmol/L carbonyl cyanide 4-(trifluoromethoxy) phenylhydrazone (an electron transport chain uncoupler), and finally 1 μmol/L rotenone (a complex III inhibitor) plus 1 μmol/L antimycin A (a complex I inhibitor). During each of the 4 stages of the assessment, the OCR was measured in 16 wells per condition at 3 different time points. All OCR data were normalized to the total protein per well, which was determined using the Bradford assay.

### RNA-Seq

RNA was extracted from pellets of 4 × 10^6^ THP-1 cells using the RNeasy mini kit (Qiagen) and any residual DNA was then removed using the DNA-free DNase treatment kit (Thermo Fisher Scientific). The RNA samples with a RNA Integrity Number > 7 were sent to AROS Applied Biotechnology A/S (Ebersberg, Germany) where the RNA-Seq was carried out. The total RNA was converted into a library of template cDNA using the Illumina TruSeq Stranded Total RNA Sample Prep kit (San Diego, Calif) and this cDNA library was then sequenced using the Illumina HiSeq 2500 machine. Reads were aligned to the hg19 (human genome version 19, Genome Reference Consortium GRCh37.p13) reference genome, annotated and normalized to produce a read per kilobase per million mapped reads for each gene. Differential gene expression between samples and conditions was determined using DESeq2 software.E3, E4 The biological significance of the changes in gene expression on cellular processes and signaling pathways was investigated using IPA (Qiagen). In IPA the differential expression data were analyzed in the context of the Ingenuity Knowledge Base, a large curated database of published observations on mammalian biology, to identify the likely upstream causes and downstream effects of any changes in gene expression.^E5^ Prior to the pathway analysis the normalized read per kilobase per million mapped data were filtered to include only genes that had >0.5 log-fold change between conditions and were significantly differentially expressed, as defined by a *P*-value adjusted for multiple comparisons using the Benjamini-Hochberg method <.05.^E6^ The assessment of the effect of the changes in gene expression on canonical signaling pathways was also filtered to only include significantly altered pathways (adjusted *P* < .05) that differed from the mean in the control sample by >2 SD (*z*-score > ±2). Gene Ontology Consortium was used to perform the gene ontology analysis. Volcano plots and heat maps were produced using R statistical software (R Foundation, Vienna, Austria). Volcano plots were produced by plotting the adjusted *P*-values against the log2-fold change of the normalized gene counts, obtained from DESeq2. The most differentially expressed genes were obtained by ordering the absolute values of the log2-fold change in descending order. The normalized counts of the top 3000 genes were then used to produce heat maps.

### Quantitative RT-PCR

RNA was extracted from pellets of 4 × 10^6^ THP-1 cells using the RNeasy mini kit and single-stranded cDNA was synthesized from this RNA using the High Capacity cDNA reverse transcription kit (Invitrogen). Following this the relative transcription of specific genes was determined by quantitative RT-PCR using the TaqMan Gene Expression Assay (Applied Biosystems) and the 7500 Fast Real Time PCR System (Thermo Fisher Scientific). The relative amount of cDNA for each specific target was determined by comparison with the control housekeeping gene *ACTB* using the difference in cycle threshold method.

### Cell surface receptor expression

The fluorescence due to the labelling of cells with phycoerythrin-conjugated antihuman TLR-4 (CD284) antibodies (both from BioLegend, San Diego, Calif) was determined using the FACSCanto II flow cytometer. The signal intensity for each receptor was then calculated.^E7^

### Statistical analysis

All experiments were carried out on a minimum of 3 biological replicates; the number of replicates used to generate the data for a specific experiment is detailed in the legend of each figure. The Shapiro-Wilk test was used to determine the normality of the data. Normally distributed data are presented as means ± SD and were analyzed using an independent *t*-test or 1-way ANOVA with Dunnett *post hoc* analysis. Nonnormal data are presented as medians ± interquartile ranges and were analyzed using the Mann-Whitney *U* test or Kruskal-Wallis analysis of variance with Dunn *post hoc* analysis. The relationship between variables was assessed by linear regression and Pearson correlation coefficient. A *P*-value of less than .05 was defined as the threshold for statistical significance.

## Supplementary data

Data file E1
